# The Antibody Assay in Suspected Autoimmune Encephalitis From Positive Rate to Test Strategies

**DOI:** 10.3389/fimmu.2022.803854

**Published:** 2022-02-23

**Authors:** Qun Deng, Ye Liu, Zhifeng Mao, Yun Chen, Yue Ping, Guoqiang Zhu, Weiqing Zhao, Xiao Hu, Hao Zhou

**Affiliations:** ^1^Department of Pediatric Neurology, Guizhou Provincial People’s Hospital, Guiyang, China; ^2^Department of Otolaryngology, Guizhou Provincial People’s Hospital, Guiyang, China; ^3^Department of Autoimmune Disease, Guangzhou KingMed Diagnostics Group Co., Ltd., Guangzhou, China; ^4^Department of Autoimmune Disease, Guizhou KingMed Diagnostics Group Co., Ltd., Guiyang, China; ^5^Department of Neurology, Guizhou Provincial People’s Hospital, Guiyang, China

**Keywords:** autoimmune encephalitis, positive rate, test strategies, anti-neuronal antibody, assay

## Abstract

**Objective:**

The aim of this study was to analyze the positive rate and test strategies of suspected autoimmune encephalitis (SAE) based on an antibody assay.

**Methods:**

Patients who were diagnosed with suspected autoimmune encephalitis in Guizhou Province between June 1, 2020, and June 30, 2021 and who had anti-neuronal autoantibodies detected by Guizhou KingMed Diagnostics Group Co., Ltd. were included in this study. The positive rate and the test strategies were analyzed based on the results of the anti-neuronal antibody assay.

**Results:**

A total of 263 patients with SAE were included, 58.2% (153/263) of whom were males, with a median age of 33 years (1-84 years). 84% (221/263) of all patients completed both serum and CSF tests. A total of 46.0% (121/263) of SAE patients received the AE-6 examination package. The antibody-positive rate was 9.9% (26/263) in the current cohort, with an observed incidence of antibody positive of 0.2 in 100,000 (26/11,570,000, 95% CI: 0.15-0.30), and the estimated incidence was 0.9 in 100,000 (95% CI: 0.84-0.95) of the total population. A total of 9 different anti-neuronal antibodies were detected. Anti-NMDAR antibody was the most common antibody in 46.2% (12/26) of subjects, 70.0% (7/10) of whom were children, followed by anti-Caspr2 antibody in 30.8% (8/26); the remaining 7 antibodies were detected in 23.1% (6/26) of the population. There were no obvious differences among age, sex or season in the positive rate of anti-neuronal antibodies. The cost of antibody testing per capita was $439.30 (SD±$195.10). The total cost of AE-14 was the highest at $48.016.81 (41.56%) among all examination packages.

**Conclusions:**

This study described the positive rate associated with AE-related anti-neuronal antibodies and test strategies in the current cohort, which provides a basis for clinicians in clinical practice.

## Introduction

Autoimmune encephalitis (AE) refers to a group of immune-mediated neurologic diseases (1). Since the initial report on antibodies in 2007, the study of AE has increased rapidly (2, 3). The prevalence (1995–2015) of AE was 0.8/100,000 in the United States (4), and a previous study demonstrated that nearly one-third of patients with encephalitis were diagnosed with AE (5). Some investigations have shown that the most common AE antibodies are the anti-N-methyl-D-aspartate receptor (NMDAR) antibody, anti-leucine-rich glioma-inactivated 1 (LGI1) antibody, anti-gamma-aminobutyric acid-B receptor (GABABR) antibody and anti-contactin-associated protein-like 2 (CASPR2) antibody, and detection rates have been reported to be 79.7%, 12.8%, 5.6%, and 1.3%, respectively (6). To date, more than twenty extracellular antibodies and forty intracellular antibodies have been identified. Currently, data on AE antibodies mainly come from Western countries, and there is a lack of clinical data on AE antibody prevalence in the Chinese population.

Studies have shown that 43% of severe AE patients require intensive care unit (ICU) treatment (5). The most common type of anti-NMDAR encephalitis has a mortality rate of up to 6% (7). Although most patients have a good prognosis, their disease burden is heavy due to the high cost of treatment. Recently, related reports have shown that the average median hospital charges per patient with AE in the U.S. exceeds 70,000 USD, and in China, it averages 86,810 USD. The cost of diagnosis and treatment of AE is a heavy burden for every family (5, 6). Early intervention can improve the prognosis of AE patients (8). Antibody detection plays a crucial role in the early diagnosis of AE (9, 10). At present, the clinical detection of autoimmune encephalitis-related antibodies is mainly carried out by the Independent Clinical Laboratory. The Independent Clinical Laboratory (ICL) is an intermediary organization that provides fair, reliable and accurate test data and test results for medical institutions. Christopher A and Wernerfelt B pointed out that third-party medical testing laboratories have developed into a large-scale industry abroad (11); as of the end of 2015, the Independent Clinical Laboratory accounted for 38% of the medical testing market in the United States, 50% in Germany, and 67% in Japan (12). The anti-neuronal antibody assay conducted by ICL in China is very common. Therefore, in this study, we carefully analyzed the positive rate and the test strategies according to the antibody assay in suspected autoimmune encephalitis provided by the ICL.

## Methods

### Study Population and Sites

Patients who were diagnosed with suspected AE according to the published AE diagnostic criteria (1, 13) in Guizhou Province between June 1, 2020, and June 30, 2021 and who had anti-neuronal antibodies detected by Guizhou KingMed Diagnostics Group Co., Ltd. were included in this study. KingMed is regarded as the pioneer and leader of the Independent Clinical Laboratories (ICL) industry and is the first ICL with both CAP and ISO15189 accreditation in China (14).

The present study sites were located in Zunyi and Guiyang district, Guizhou Province, Southwest China. Guizhou is a relatively poor and economically undeveloped city in China with a population of 38.56 million. The study sites are the most developed cities in Guizhou Province. There are more than 11.57 million inhabitants in Guiyang, and Zunyi make up approximately 30% of the population, which is the economical and medical center in Guizhou Province. A total of approximately 1,050 cases were diagnosed with suspected AE, and anti-neuronal antibodies were detected by LCL in two districts. The data came from six medical centers, which have qualifications for the diagnosis of AE in the study sites.

### Data Collection

All data were collected with a standard sheet. Demographic characteristics, including age, sex and the time of antibody investigation, were recorded. Antibody investigation methods; sample types, including serum and cerebrospinal fluid (CSF); positive results, antibody titers; methodology; and the cost of antibody investigation were recorded and assessed.

### Assay for AE Antibodies

Antibodies of SAE were detected in both serum and CSF at Guizhou KingMed Diagnostics Group Co., Ltd. Regarding the antibody investigation strategy, seven strategies (**Table 1**) were used in all patients, which included strategies to detect intracellular and extracellular antibodies. HEK293 cells were seeded on 96-well plates and transfected with anti-neuronal antibodies for 36 hours. The cells were fixed with 4% paraformaldehyde for 20 minutes for antibody testing. The cells were incubated for 2 hours at room temperature with undiluted cerebrospinal fluid (CSF) and serum diluted 1:10 in phosphate buffered saline (PBS)-0.1% goat serum. The cells were incubated with Tween-20 three times and goat anti-human IgG (1:500; Thermo Scientific) for 30 minutes and washed again in PBS-0.1% Tween-20. Then, an immunofluorescence microscope was used for evaluation. The samples with positive results were tested at least twice. Two independent masked assessors classify each sample as positive or negative based on the surface immunofluorescence intensity, which is directly compared with nontransfected cells and control samples. Once verified, the positive samples were then serially diluted from 1:10 to 1:1000 to determine the titer. The final titer was defined as the dilution value of the sample whose specific fluorescence was almost clearly identifiable and expressed as the corresponding dilution value (15).

**Table 1:** Different detection strategies for anti-neuronal antibodies in Guizhou KingMed.

Strategies	Antibodies
AE-6	NMDAR, AMPA1, AMPA2, LGI1, CASPR2, GABAB
AE-8	NMDAR, AMPA1, AMPA2, LGI1, CASPR2, GABAB, IgLON5, DPPX
AE-12	NMDAR, AMPA1, AMPA2, LGI1, CASPR2, GABAB, IgLON5, DPPX, GlyR1, DRD2, MGluR5, GAD65
AE-14	NMDAR, AMPA1, AMPA2, LGI1, CASPR2, GABAB, IgLON5, DPPX, GlyR1, DRD2, MGluR5, GAD65, MGluR1, Neurexin-3α
AE-20	NMDAR, AMPA1, AMPA2, LGI1, CASPR2, GABAB, GABAA, IgLON5, DPPX, GlyR1, DRD2, MGluR5, GAD65, MGluR1, Neurexin-3α, gAchR, KLHL11, AQP4, MOG, GFAP
PNS-11	Hu, Yo, Ri, CV2, Ma2, AMphiphysin, Tr(DNER), GAD65, Ma1, Zic4, SOX1
PNS-14	Hu, Yo, Ri, CV2, Ma2, AMphiphysin, Tr(DNER), GAD65, Ma1, Zic4, SOX1, PKCγ, Titin, Recoverin

### Statistical Analysis

GraphPad Prism 9.0 (GraphPad Software Inc., San Diego, CA) and SPSS 26.0 (SPSS Inc., Chicago, IL) were used for the statistical analyses. The two incidences of AE were calculated: (1) the observed incidence, i.e., a proportion based on antibody-positive AE, and (2) an estimated incidence according to the total of approximately 104 cases (1050*0.099) were diagnosed with suspected AE, and antibodies of SAE were detected in the two districts. The number of antibody positivity was estimated based on the positive rate of the current cohort. The denominator used for incidence calculations (11.57 million) was determined by the total population of Guiyang and Zunyi. Categorical variables are described as percentages. The chi-squared test was used to compare differences among sexes, ages, sample types and seasons. Fisher’s exact test was used to compare differences among age groups and examination packages. An independent-sample t test was employed to compare differences among the testing costs of CSF samples and serum samples. In addition, *P<0.05* indicated a significant difference.

## Results

### Basic Information of this Cohort

**Figure 1:**
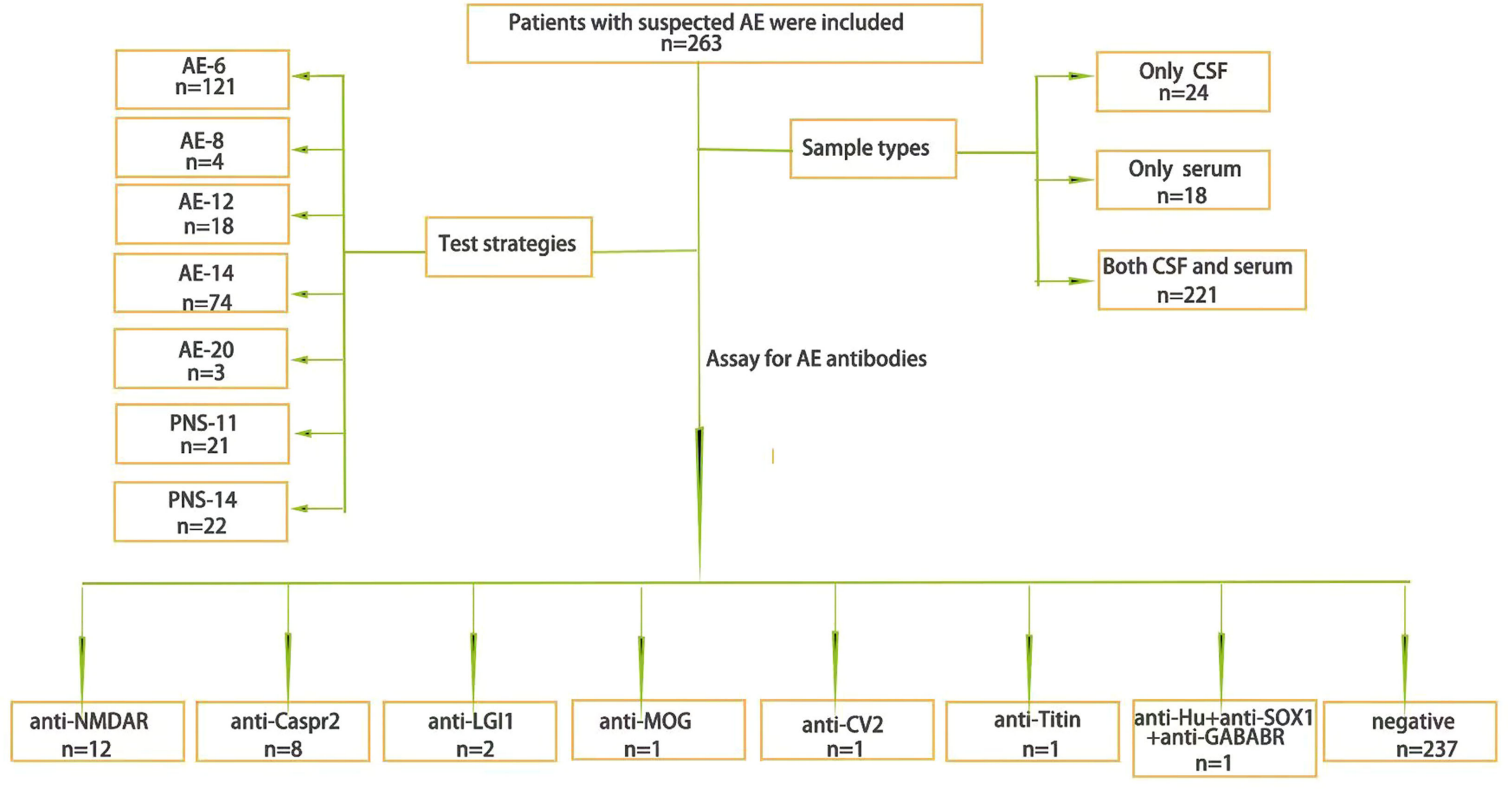
The general situation. The general situation of AE antibody testing samples and test strategies and the results.

A total of 263 patients with suspected AE were included, 58.2% (153/263) of whom were males and 33.5% (88/263) of whom were children (≦18 years old), with a median age of 33 years (1-84 years). A total of 221 patients completed both serum and CSF tests; 24 patients completed only CSF testing, and 18 patients completed only serological testing (**Figure 1**). Finally, a total of 26 patients were antibody-positive, 92.30% (24/26) of cases with varying degrees of language disorders and memory changes, 84.62% (22/26) present with psychiatric symptoms, 73.08% (19/26) have showed dyskinesia and consciousness disorders, the details clinical characteristics of different antibodies see the **Table S1**.

### Incidence of Antibody-Positive AE

The positive rate was 9.9% (26/263) in the current cohort, with an observed incidence of antibody positive of 0.2 in 100,000 patients (26/11,570,000, 95% CI: 0.15-0.30). A total of 104 cases (0.099*1,050) were estimated according to the positive rate of the current cohort. The estimated incidence was 0.9 in 100,000 (104/11,570,000, 95% CI: 0.84-0.95) of the total population.

### Status of AE Antibodies

**Figure 2:**
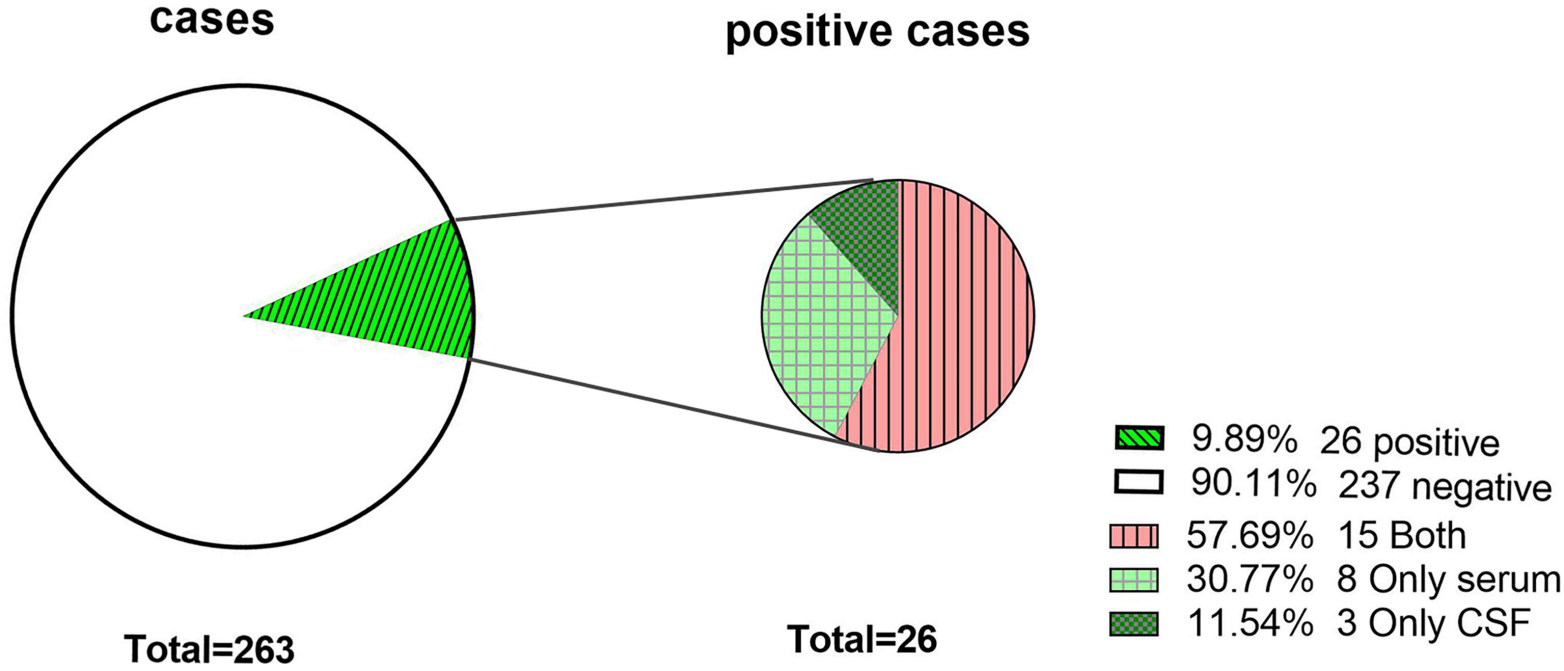
The antibody-positive rate of the study population. Antibodies were detected in both CSF and serum. Only serum, antibodies were detected in only serum. Only CSF, antibodies were detected in only CSF.

A total of 57.7% (15/26) were detected in both CSF and serum, 30.8% (8/26) were detected in only serum, and 11.5% (3/26) were detected in only CSF. The positive rate of serum detection was slightly higher than that of CSF detection (*X^2^ = 0.81, P=0.37*) (**Figure 2****).**

**Figure 3:**
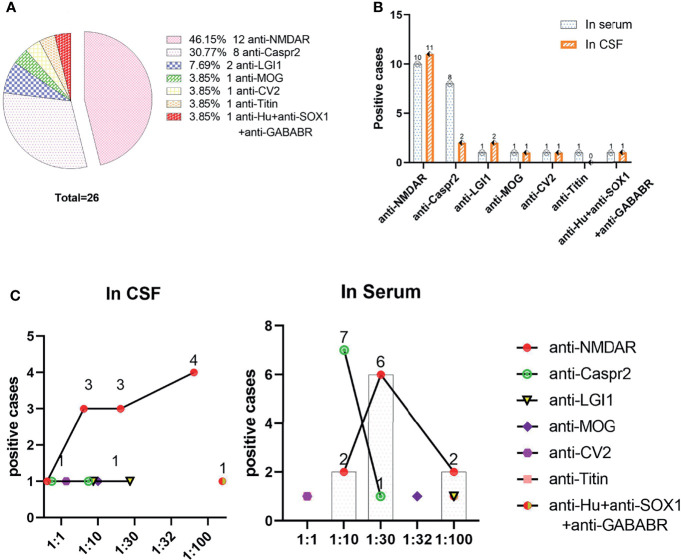
Distributions of positive antibodies and antibody titers. **(A)** Distributions of positive antibodies; **(B)** Detection of positive antibodies in serum and/or CSF; **(C)** Corresponding titers of various antibodies in serum and CSF.

**Figure 3** shows that the anti-NMDAR antibody was the most common, at 46.2% (12/26) in the total population and 70.0% (7/10) in the pediatric population, followed by anti-Caspr2, at 30.8% (8/26). Among the patients with positive anti-Caspr2 antibody, only 25.0% (2/8) were detected in CSF, and the remaining 7 antibodies (anti-LgI1, anti-MOG, anti-CV2, anti-titin, anti-Hu, anti-SOX1 and anti-GABABR antibodies) were detected in 23.0% (6/26) of the population. Anti-Titin antibodies were detected in only serum. The antibody titers of serum and CSF were 1:1 to 1:100.The antibody titer of 1:100 anti-NMDAR antibody in CSF vs. serum was 36.36% (4/11) vs. 20% (2/10), X^2^ = 0.119, P=0.730. The titer of anti-Caspr2 antibody in serum was mainly 1:10 87.5% (7/8).

**Figure 4:**
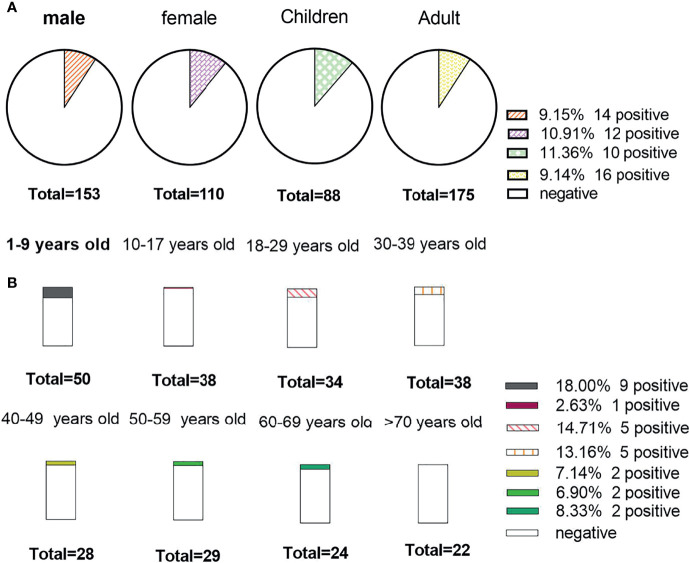
The positive rate of the study data stratified by sex and age. **(A)** Detection results of antibody among sexes, children and adults; **(B)** The positive rate among different age groups.

**Figure 5:**
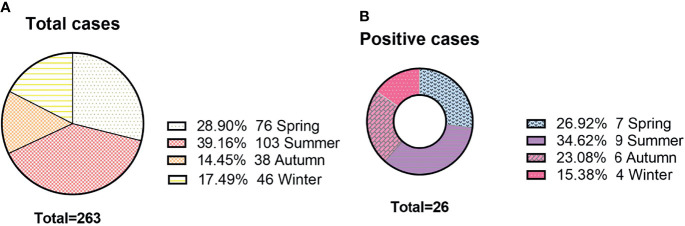
The seasonal distributions of antibody detection. **(A)** The seasonal distributions of cases; **(B)** The number of positive cases among different seasons.

There was no obvious difference between age and sex in the positive rate of the AE antibody assay (11.4% vs. 9.1% for age, X^2^ = *0.324, P=0.569*; 9.2% vs. 10.9% for sex, X^2^ = 0.22, *P=0.*637). In **Figure 4**, we can see that the positive rate of AE antibody had the highest peak in those aged 1-9 years (34.6%, 9/26) and was lowest in those aged 10-17 years, at 3.8% (1/26). However, there was no significant difference between the various age groups (*X^2^ = 9.618, P=0.175*). A total of 39.16% (103/263) of clinical samples were detected in summer. The positive rate of antibody was the highest in summer (34.62%, 9/26). The results revealed no significant seasonal variations in the positive rate of AE antibody (*X^2^ = 1.774, P=0.628*) (**Figure 5**).

**Figure 6:**
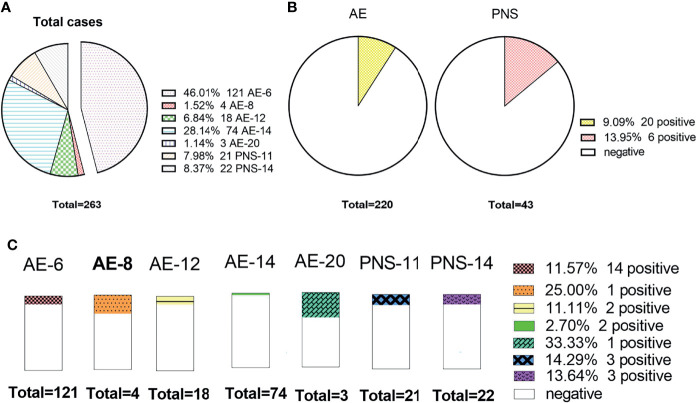
The status of AE antibody among different package items. **(A)** The number of cases detected by different packages; **(B)** The comparison of the positive rate between the AE and paraneoplastic syndrome packages; **(C)** The distributions of the positive rate among different package items.

**Figure 6** shows that 46.0% (121/263) received the AE-6 examination package, but AE-20 only 1.14% (3/263). The positive rate of PNS (13.95%) antibodies was higher than that of AE (9.09%) antibodies, and there was no obvious difference among them (*X^2^ = 0.487, P=0.485*). AE-8 and AE-20 were not included in this study because there were very few cases. The positive rate of paraneoplastic syndrome packages (PNS-11) was the highest, at 14.29% (3/21), and that of AE-14 was the lowest, at 2.70% (2/74). There was no significant difference between the various examination packages (*X^2^ = 10.882, P=0.059*).

### Testing Costs

**Figure 7:**
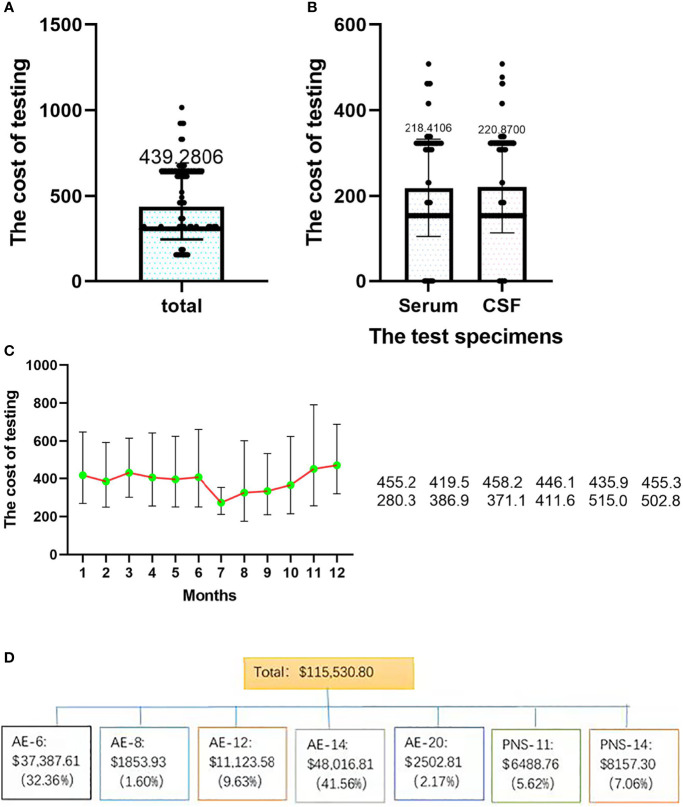
The distributions of testing costs among different samples and seasons. **(A)** Total testing cost; **(B)** the costs of different samples; **(C)** the costs in different seasons. **(D)** the costs of different test strategies.

The total testing cost was $115,530.80, the total testing cost of CSF samples was $58,088.80, and the total testing cost of serum samples was $57,442.00. From **Figure 7**, it can be concluded that the cost per capita for testing was $439.30 (SD±$195.10), the cost per capita for CSF and serum samples was $220.90 (SD±$114.20) and $218.40 (SD±$108.10), and the average cost per person was $280.30 (SD±$58.90) and $386.90 (SD±$262.30), respectively. There was no significant difference between serum and CSF (*t=0.254, P=0.8*). The total cost of AE-14 was the highest at $48,016.81 (41.56%). AE-6 was $37,387.61 (32.36%), and PNS-11 was $6488.76 (5.62%) (**Figure 7**).

## Discussion

AE comprises a group of severe immune-mediated diseases of the brain and is emerging as a relatively common cause of encephalitis worldwide. However, existing evidence reveals that there are no exact epidemiological data in developing countries. A total of 236 patients with suspected AE were analyzed retrospectively with the anti-neuronal antibody assay. The results demonstrated that the total positive rate was 9.9%, and the observed incidence of antibody positivity was 0.2 in 100,000 patients (95% CI: 0.15-0.30). The estimated incidence was 0.9 in 100,000 (95% CI: 0.84-0.95), and the anti-NMDAR antibody was the most common (46.2%) among the 9 AE antibodies detected in CSF or serum. The antibody titer of CSF and serum ranges from 1:1 to 1:100. There were no obvious differences among age, sex or season in the positive rate of anti-neuronal antibodies. The cost of antibody testing per capita was $439.30.

The detection of antibodies is extremely important for the diagnosis and treatment of AE (16–18). Early diagnosis, early intervention and early treatment of AE can significantly improve the survival rate and prognosis (5, 18). The total positivity rate was 9.9% in the current cohort, which is similar to the 12.7% reported in previous studies (5). A total of 30.8% of all positive cases were detected in only serum, but only 11.5% were detected in CSF. This indicates that the total positive detection rate in serum is higher than that in CSF. The main clinical symptoms of encephalitis with different antibody types are similar, but treatment plans and prognoses are different (19). For example, anti-Caspr2 indicates thymoma, and anti-GABAB indicates small cell lung cancer (19–24). Paraneoplastic syndromes are usually relatively insidious and asymptomatic, so antibody testing is needed to assist in diagnosis (25). Our study showed that the anti-NMDR antibody was the most common. A previous study also found that anti-NMDR encephalitis was the most common AE antibody (26). It is worth noting that anti-IGI1 was detected in both CSF and serum, and anti-Hu IgG was detected in only serum in this study, which is different from previous studies (17, 26, 27). Whether these antibodies with a very low positive rate are detected in CSF or serum, more research is needed to draw corresponding conclusions. The results showed that the antibody titers of serum and CSF were 1:1 to 1:100. A previous study revealed that the titers and types of antibodies also changed during the course of disease progression (28–30), which can be used for disease assessment and prognosis.

The estimated incidence was 0.9 in 100,000 patients in our study, which was similar to that in Divyanshu Dubey et al. reported that the incidence rate (1995-2015) of AE was 0.8/100,000 person-years in United States (4). The results demonstrated that there was no difference in the positive rate between females and males, which is different from previous studies (25). However, the positive rate in children (11.36%) was higher than that in adults (9.14%). Among all age groups, the positive rates of antibodies in serum and CSF were highest in the age group of 1-9 years, which is consistent with the results of previous studies indicating that AE mainly occurs in children and young people (26). It was found that 39.16% of people submitted samples for inspection in summer. Therefore, encephalitis symptoms are more common during this time period. In addition, the highest antibody positive rate was in spring. To determine whether AE antibodies are affected by season, more sample data are needed for statistical research.

The testing of antibodies in China mainly relies on the ICL; in general, the LCL will design different antibody tested packages to fit different requirements. People received the AE-6 inspection package most often (46.0%), but the highest positive rate was for PNS-11 (14.29%), and the positive rate of AE-14 was the lowest (2.7%) in the current study. Interestingly, we found that when the selected items increased, the detection positive rate decreased. This may be related to the patient’s clinical symptoms. When the symptoms are relatively mild, clinicians will tend to choose the most common inspection packages. With the widespread application of MOG antibody detection, reports of encephalitis associated with MOG antibodies are increasing (31, 32). In the inspection package provided by the ICL, only AE-20 contained the antibody, and 98.9% (260/263) were not tested for anti-MOG antibody. However, anti-MOG antibody encephalitis is easily missed. The current low positive rate of MOG antibodies is related to our inactive inspection. This requires more data to confirm.

Regarding AE vs. PNS (9.09% vs. 13.95%), PNS related to neuronal surface antibodies was more common. Ninety-two patients over 45 years old were tested for antibodies to paraneoplastic syndrome. That is, 89.13% (82/92) of patients who were ≥45 years of age did not. However, paraneoplastic syndrome mainly occurs in middle-aged and elderly people. Therefore, clinicians need to consider the common types of antibodies of different ages when choosing inspection packages. The diagnosis and treatment of AE have imposed a great burden on people (5, 6). According to our research data, the average cost for antibody testing was $439.30. The overall cost of testing is high, but the positive rate is only 9.9%. The total cost of AE-14 was the highest among all examination packages, but the positive rate was only 2.70%. There is a great waste of resources, which brings some reflection for clinicians. When submitting specimens, they should strictly control the indicators for submitting specimens to improve the positive rate and reduce the medical cost.

## Limitations

This study performed statistical and test strategy analyses of antibody detection results. Although detailed analysis was carried out for various aspects, there are several limitations to this study. First, the sample size was relatively small, and the data were obtained from only one clinical laboratory; therefore, the results explain only the positive rate and test strategies of the detection of AE-related antibodies in the current cohort. Second, we described the estimated antibody-positive incidence of AE in this study. Nevertheless, the results may not be generalizable to the total AE cohort. The inclusion of antibody-negative children should be considered in future multicenter epidemiological studies when technically feasible. Third, no positive antibodies were found in serum or CSF in patients over 70 years old, which may be due to the small sample size. Finally, research mainly focuses on AE antibody data, and clinical data to analyze the research results considering all aspects are lacking.

## Conclusions

The clinical manifestations and diagnosis of AE are extremely complicated, and the continuous development of inspection technology for detection antibodies is beneficial to the diagnosis and prognosis of diseases and has important guiding significance. In our study, we first described the positive rate and test strategies of the detection of AE-related anti-neuronal antibodies in the current cohort, with a total positive rate of 9.9%, and the estimated incidence was 0.9 in 100,000 (95% CI: 0.84-0.95) of the total population. A total of 9 different anti-neuronal antibodies were detected, and the anti-NMDAR antibody was the most common. The results provide a basis for clinicians to see AE in clinical practice.

## Data Availability Statement

The original contributions presented in the study are included in the article/**Supplementary Material**. Further inquiries can be directed to the corresponding author.

## Ethics Statement

The studies involving human participants were reviewed and approved by Guizhou Provincial People’s Hospital. Written informed consent to participate in this study was provided by the participants’ legal guardian/next of kin. Written informed consent was obtained from the individual(s), and minor(s)’ legal guardian/next of kin, for the publication of any potentially identifiable images or data included in this article.

## Author Contributions

HZ conceived of the study. QD, YL, and ZM contributed to the analysis, synthesis and interpretation of the results and wrote the manuscript. YC, YP, GZ, WZ, and XH contributed to the data collection. All authors contributed to the preparation of the manuscript. All authors contributed to the article and approved the submitted version.

## Funding

This project was supported by the National Natural Science Foundation of China (NSFC, 81860280).

## Conflict of Interest

Author ZM was employed by Guangzhou KingMed Diagnostics Group Co., Ltd. GZ was employed by Guizhou KingMed Diagnostics Group Co., Ltd.

The remaining authors declare that the research was conducted in the absence of any commercial or financial relationships that could be construed as a potential conflict of interest.

## Publisher’s Note

All claims expressed in this article are solely those of the authors and do not necessarily represent those of their affiliated organizations, or those of the publisher, the editors and the reviewers. Any product that may be evaluated in this article, or claim that may be made by its manufacturer, is not guaranteed or endorsed by the publisher.
