# Supplementary material for: The Antibody Assay in Suspected Autoimmune Encephalitis From Positive Rate to Test Strategies
Source: Front Immunol. 2022 Feb 23;13:803854. doi: 10.3389/fimmu.2022.803854 (PMC8904559; doi:10.3389/fimmu.2022.803854)
Supplement: Supplementary file 1 [file Table_1.docx]

**Table S1 The clinical characteristics of different antibodis positive patients(N, Median(IQR), %)**

| **Characteristics** | **Total** |  | **Antibody type** | |
| --- | --- | --- | --- | --- |
|  |  | **NMDR** | **Caspr2** | **Other types** |
| **Number** | **26** | **12** | **8** | **6** |
| **Age (years), median (IQR)** | **21.5(6.75-40)** | **9(4.25-26.25)** | **35.5(17.5-49)** | **36(17.5-65.25)** |
| **Gender, female : male** | **12:14** | **5:7** | **4:4** | **3:3** |
| **Clinical features, n (%)** |  |  |  |  |
| Seizure  Memory dysfunction  Psychiatric symptom  Consciouness  Language problem  Dyskinesia  Gait instability and ataxia  Brainstem dysfunction  Weakness | 9(34.62)  24(92.31)  22(84.62)  19(73.08)  24(92.31)  19(73.08)  8(30.77)  10(38.46)  7(26.92) | 4(33.33)  11(91.67)  12(100.00)  11(91.67)  11(91.67)  11(91.67)  2(16.67)  4(33.33)  5(41.67) | 1(12.50)  8(100.00)  8(100.00)  7(87.50)  8(100.00)  7(87.50)  5(62.50)  5(62.50)  1(12.50) | 4(66.67)  5(83.33)  2(33.33)  1(16.67)  5(83.33)  1(16.67)  1(16.67)  1(16.67)  1(16.67) |

Note: IQR, interquartile range
